# Rheumatoid Cachexia Revisited: A Metabolic Co-Morbidity in Rheumatoid Arthritis

**DOI:** 10.3389/fnut.2014.00020

**Published:** 2014-11-24

**Authors:** Kayo Masuko

**Affiliations:** ^1^Graduate School of Nutritional Science, Sagami Women’s University, Sagamihara-shi, Japan

**Keywords:** rheumatoid cachexia, cachexia, sarcopenia, inflammation, rheumatoid arthritis

## Abstract

Rheumatoid arthritis (RA) is a chronic inflammatory disease in which pro-inflammatory cytokines, including tumor necrosis factor (TNF)-α, play a crucial role. The chronic inflammation, combined with reduced physical activity, leads to muscle wasting whereas fat mass would be maintained; the resulting abnormal metabolic state is described as rheumatoid cachexia. Since the loss of muscle volume would be compensated by the increased fat mass, body mass index (BMI) is reported not to reflect the nutritional status in RA patients. The implication of rheumatoid cachexia for cardiovascular risk and clinical prognosis is not clearly understood, however, adequate control of disease activity in combination with appropriate physical exercise could be the most important strategy to control rheumatoid cachexia and related metabolic problems.

## Introduction

Recent advances in the management of rheumatoid arthritis (RA) have reduced the frequency of so-called “cachexia” in RA patients. In such “classic” cachexia, patients often look emaciated, under a malignant or chronic inflammatory condition with appetite loss and protein degradation. However, in RA, persistent inflammation may induce muscular wasting without thinning; the muscle loss in RA would be associated with normal or even obese appearance. The condition has been named “rheumatoid cachexia.” Although its impact on morbidity and mortality has been suggested, the significance of rheumatoid cachexia is not fully understood in part because of the lack of specific symptoms.

The present article reviews the current concept of rheumatoid cachexia, including the confusion regarding its clinical definition, and highlights the potential importance of physical exercise and nutritional assessment in the therapeutic strategy against RA.

## Definition of Cachexia and Related Conditions

### Cachexia (in classic meaning)

Cachexia is manifested by unintended severe weight loss and muscle wasting, and can be defined as “a multifactorial syndrome characterized by severe weight, fat and muscle loss, and increased protein catabolism due to underlying disease(s)” (1, 2). The term “cachexia” originates from the ancient Greek for “bad condition,” and often occurs in patients with advanced malignancy (cancer cachexia); chronic heart failure (cardiac cachexia); chronic renal failure; chronic obstructive pulmonary disease (COPD); acquired immunodeficiency syndrome (AIDS); and many other chronic diseases, including advanced RA.

Clinically, cachexia can be detected by assessment of body composition (i.e., measurement of muscle mass and fat mass) generally using bioimpedance analysis (BIA) or dual-energy X-ray absorptiometry (DEXA). Muscle mass can be measured as fat-free mass (FFM) by BIA, or lean body mass (LBM) by DEXA; values reflect the estimated mass of skeletal muscle and its metabolic activity.

Since cachexia is known to be a major cause of increased mortality, “early and effective interventions” are needed to prevent the development of fatal metabolic abnormalities (1). To this end, efforts have been made to develop and share a consensus on the clinical definition of cachexia, including diagnostic criteria. In 2005, the Special Interest Group (SIG) on cachexia–anorexia in chronic wasting diseases was created in the European Society for Clinical Nutrition and Metabolism (ESPEN) (2). The SIG developed a clinical definition of cachexia (Table 1), and to prevent delayed diagnosis and intervention, introduced the term “pre-cachexia.” Pre-cachexia was defined as the simultaneous presence of the following four features; an underlying chronic disease; unintentional body weight (BW) loss (≤5%) [cf: (≥) in cachexia] during the last 6 months; chronic or recurrent systemic inflammation; and anorexia or symptoms of appetite loss (2).

**Table 1:** **Clinical criteria for cachexia, rheumatoid cachexia, and sarcopenia**.

Criteria	Classic Cachexia	Rheumatoid Cachexia		Sarcopenia	
Reference	ESPEN-SIGs (2)	Engvall (20)	Elkan (19)	ESPEN-SIGs (2)	EWGSOP (3)

Concept	Loss of body weight; loss of both lean and fat mass; loss of skeletal muscle mass	Decreased FFM and increased fat mass		Loss of skeletal muscle mass and strength	

Underlying chronic disease	+	Rheumatoid Arthritis		±	

Body weight/BMI	[*] Unintentional weight loss (≥5%) during the last 12 months, or BMI <20 kg/m^2^	→or ↑		

Muscle strength	[1] Decreased muscle strength				[1] Decreased muscle strength, e.g., low handgrip strength (<30 kg in men, or <20 kg in women)

Fatigue	[2] Fatigue				

Appetite (continued)	[3] Anorexia or anorexia-related symptoms				

Body composition	[4] Low FFM (lowest 10%)	FFMI <10th percentile and FMI >25th percentile	FFMI <25th percentile and FMI >50th percentile	[*] Low muscle mass; e.g., ≥2 SD below the mean in young adults of the same sex and ethnic background	[*] Low muscle mass; e.g., ≥2 SD below the mean in young adults

Role of cytokines	[5] Evidence of cytokine excess: (1) CRP >0.5 mg/dL or IL-6 >4.0 pg/ml, (2) Hb <12 g/dl, (3) Alb <3.2 g/dL	Dominance of inflammatory cytokines(in particular TNFα and IL-6) due toinflammatory arthritis			

Fat mass	↓	→or ↑		→(or ↑in sarcopenic obesity)	

Physical performance				[1] Low gait speed (≤0.8 m/s in the 4-m walking test); [2] reduced performance test (used fro the CGA)	[2] E.g., Low usual gait speed (≤ 0.8 m/s in the 4-m walking test) and/or [2] Low physical performance, SPPB ≤8

Diagnostic criteria	Defined as cachexia if [*] plus ≥3 among [1] to [5] are fulfilled	No consensus on the cut-off values		Defined as sarcopenia if [*] plus [1] or [2] are fulfilled	Defined as sarcopenia if [*] plus [1] or either of [2] are fulfilled

[*] indicates necessary condition for the diagnosis of “classic condition”.

*[1–5] indicates other conditions used for the diagnosis. (as described in “Diagnostic Criteria” in the Table)*.

*Modified from Muscaritoli et al. (2). EWGSOP, European Working Group on Sarcopenia in Older People: FFM, fat-free mass; FFMI, fat-free mass index; CGA, Comprehensive Geriatric Assessment; SPPB, short physical performance battery, Hb, hemoglobin; Alb, albumin; SD, standard deviation*.

Both cachexia and pre-cachexia are frequently associated with inflammation; an imbalance between pro-inflammatory (TNF-α, Interleukin (IL)-6, and IL-1) and anti-inflammatory (e.g., IL-4, IL-12, IL-15) cytokines is considered to contribute to the pathogenesis (2, 3) (Table 1).

### Sarcopenia

Sarcopenia is defined as “a syndrome characterized by progressive and generalized loss of skeletal muscle mass and strength with a risk of adverse outcomes such as physical disability, poor quality of life, and death” (3). Primary sarcopenia is usually due to aging, while secondary sarcopenia occurs due to chronic illness, immobility, and/or malnutrition. Although sarcopenia is generally an age-related condition, it can develop at a younger age, similar to other typically age-related conditions such as dementia and osteoporosis (3). The European Working Group on Sarcopenia in Older People (EWGSOP) developed a clinical definition, and consensus diagnostic criteria for sarcopenia (Table 1). According to this definition, most cachectic patients are sarcopenic, but sarcopenic patients are not necessarily cachectic (3).

In sarcopenia, the decreased muscle mass may be associated with normal, or even increased fat mass, a condition termed “sarcopenic obesity.” It has been reported that fat infiltration into muscle would lower muscle quality (3).

### Rheumatoid cachexia

Rheumatoid arthritis is an autoimmune disease that affects multiple synovial joints, and is associated with multiple extra-articular manifestations. Synovial fibroblasts and macrophages, activated by an unknown trigger, proliferate and produce a plethora of pro-inflammatory cytokines and catabolic proteases that would degrade bone and cartilage.

Due to the chronic inflammation, RA patients often exert abnormal metabolism, including insulin resistance (4, 5). Further, patients with refractory RA or in advanced stage of RA have been recognized to be at risk of cachexia. To this concern, for example, apparent BW loss (≥5% in past 12 months) was in 1.0%, whereas anorexia was shown in 5.8 (when assessed with visual analog scale) to 10.7% (when assessed with the functional assessment of anorexia/cachexia therapy), in RA patients; overall, it has been estimated that patients with “classic” cachexia (Table 1) would be approximately 1.0% in RA patients (6). In addition, to support the inflammation-induced cachectic condition, an animal model of RA had decreased daily food intake (anorexia) and BW loss, accompanied with muscle wasting (7). Nevertheless, RA patients do not necessarily lose BW and often show normal range of BMI; thus may not fit into the diagnostic criteria of “cachexia” (as indicated in Table 1). Instead, a unique abnormal metabolic state, i.e., rheumatoid cachexia, has been proposed.

Specifically, in early studies, Roubenoff et al. documented decreased body cell mass (BCM) and increased resting energy expenditure (REE) in patients with RA, suggesting involvement of cytokine-driven hyper-metabolism (8, 9). The authors concluded that “cytokine production in RA is associated with altered energy metabolism and intake, despite a theoretically adequate diet,” and defined the condition of reduced BCM without obvious fat loss as “rheumatoid cachexia” (8, 10). Thus, long-standing inflammation in RA leads to a state of hyper-metabolism resulting in muscle wasting (11).

A number of studies followed to assess the clinical implications of altered body composition in RA [Table S1 in Supplementary Material; earlier studies are summarized in a review by Summers et al. (12)]. Most reports documented that muscle mass in RA patients was decreased, whereas the fat mass remained normal, or was increased. REE was normal or increased, with some studies showing a correlation between REE and disease activity (11, 13, 14). On the other hand, total energy expenditure (TEE) was reported to be lower than healthy controls (15), that is attributed to a reduced physical activity (16, 17).

Overall, the combined effect (i.e., loss of muscle mass and normal fat deposition) would keep BW within a normal range (18). This condition can be defined as rheumatoid cachexia, or rheumatoid cachectic obesity (8, 10, 13), which is consistent with reports that BMI is not appropriate to detect rheumatoid cachexia (19, 21). In addition, the widely used mini nutritional assessment (MNA) was not a powerful tool for detection of rheumatoid cachexia (19, 21).

To date, there remain no consensus clinical criteria for the diagnosis of rheumatoid cachexia. Van Bokhorst-de van der Schueren et al. (6) assessed the feasibility of using the ESPEN SIG definition of cachexia in a sample of 103 RA patients, and concluded that the definition was inappropriate, since reductions in BW and appetite are not apparent in RA. Engvall et al. (20) and Elkan (22) defined rheumatoid cachexia using different criteria (Table 1), and estimated the frequency of rheumatoid cachexia as 38%, or 18% of women and 21% of men, respectively. Effort is therefore needed to establish the diagnostic criteria for rheumatoid cachexia (18).

## Pathogenesis of Rheumatoid Cachexia

A variety of factors are involved in the pathogenesis of classic cachexia, including suppression of the growth hormone (GH)/insulin-like growth factor (IGF) system; testosterone deficiency; and an excess of myostatin and glucocorticoids (16). In rheumatoid cachexia, relative excess of pro-inflammatory cytokines is considered to be the central feature (9). Pro-inflammatory cytokines including TNFα, IL-1β, IL-6, and IFN-γ, the key players of synovitis and extra-articular manifestations, may activate the nuclear factor κB and lead to an increase in muscle proteolysis via the ubiquitin-proteasome pathway (16). Overexpression of IL-1β would also induce anorexia and exacerbate muscle loss (9). Further, use of glucocorticoid as a pharmacological treatment against symptoms of RA might aggravate the rheumatoid cachexia. More specifically, glucocorticoids have been shown to induce muscle atrophy (steroid myopathy) through activation of the transcription factor FOXO or repression of mTOR signaling, leading to protein catabolism (23).

The GH-IGF axis may also be an important contributor, although controversy exists (24). In an animal model of chronic inflammatory arthritis, Ibanez de Caceres et al. reported a decrease in BW and reduced levels of circulating IGF-1; administration of recombinant GH was associated with increased BW gain without an increase in food intake, and increased levels of IGF-1 (25). On the other hand, in the model, arthritis was also associated with increased expression of the cyclooxygenase (COX)-2 gene; and administration of non-steroidal anti-inflammatory drugs (NSAIDS) reversed the COX’s inhibitory effect of arthritis on BW, increased liver IGF-1 levels, and enhanced the expression of ubiquitin-ligating enzymes. The authors suggested that COX-2 expression was responsible for the arthritis-induced cachexia, through modification of the GH-IGF axis and the ubiquitin-proteasome pathways (25). The use of recombinant GH in cachectic humans has been also approved for use in patients with AIDS (26); however, its efficacy in patients with RA is unclear.

## Therapeutic Strategy Against Rheumatoid Cachexia

The consequences of rheumatoid cachexia go beyond muscle wasting and fat deposition; while any impact on cardiovascular risk remains controversial (18), it is suggested that rheumatoid cachexia exacerbates RA-associated disability and morbidity (17, 27), probably leading to sarcopenia. The muscle volume of RA patients is reported to correlate with RA activity, not with dietary intake (14, 17). Further, even in RA patients with a high dietary intake of saturated fat, there was no correlation between fatty acid intake and rheumatoid cachexia (22). The most important strategy against metabolic abnormalities in RA may be the appropriate suppression of disease activity, for example, using anti-cytokine therapy and physical exercise (28).

### Disease-modifying anti-rheumatic drugs

Rheumatoid arthritis patients typically start anti-rheumatic therapy using conventional disease-modifying anti-rheumatic drugs (DMARDs), which may include methotrexate (MTX) as an anchor drug, and sulfasalazine (SSZ). These agents are effective in the suppression of disease activity, particularly when used in combination. The use of DMARDs in early RA is also reported to improve insulin resistance and dyslipidemia (5, 29, 30); this is likely due to their disease-modifying effects, rather than a direct effect on metabolism, since neither MTX nor azathioprine, when used alongside NSAIDs, was shown to affect energy metabolism in RA patients (9). Indeed, insulin resistance in RA patients was shown to be higher than that observed in patients with systemic lupus erythematosus, and was correlated with serum levels of IL-6, TNFα, and C-reactive protein (CRP) (30, 31). Perhaps surprisingly, use of predonisolone in RA patients had no correlation with BCM (Table S1 in Supplementary Material) (9).

### Anti-cytokine therapies

Over the last decade, data have accumulated regarding the long-term use of anti-TNF agents in RA patients. While solid evidence exists for their anti-arthritic efficacy, few reports indicate whether anti-TNF agents could improve rheumatoid cachexia.

Marcora et al. (32) analyzed change in body composition in 26 RA patients after 24 weeks treatment using etanercept (ETN) or MTX, and failed to find significant benefit of ETN compared with MTX. However, in a subgroup analysis of patients who gained BW during treatment, the ETN-treated patients gained a greater proportion of FFM (in the composition of the BW gained) than the MTX-treated group. Similarly, Elkan et al. (21) found no correlation between use of anti-TNF agents and body composition.

After a longer period of observation (1 year), Chen et al. (33) found that twice weekly administration of ETN was associated with increased BW, and increased serum levels of glucose-dependent insulinotropic polypeptide (GIP), leptin, and ghrelin compared with treatment with DMARDs. Further, a recent prospective study (34) involving a 2-year period of TNFα inhibition, demonstrated an increase in BW, BMI, total fat mass, and fat in the android (truncal) region; visceral fat also increased, without any change in serum leptin and adiponectin levels. The authors concluded that long-term TNF inhibition was associated with increased fat mass, with a shift to the android-visceral region, raising questions regarding the influence of TNF inhibition on cardiovascular risk. This association is surprising, given that anti-TNF therapy is reported to improve insulin resistance in RA patients (30, 35–38).

IL-6 is another important pathogenic cytokine in both classic and rheumatoid cachexia. Serum IL-6 levels in RA patients are higher than those of healthy individuals, and are reported to correlate with a high REE (11, 14). However, it remains unclear whether IL-6 levels correlate with muscle mass and/or visceral fat mass in RA (11). Further studies are needed to clarify the involvement of IL-6 in muscle degradation, and the potential effect of the anti-rheumatic agent tocilizmab, a widely used monoclonal antibody against the IL-6 receptor, in rheumatoid cachexia.

### Resistance training and endurance training

Summers et al. suggested that alongside anti-cytokine therapy, adjunctive anabolic therapy, such as high-intensity progressive resistance training (PRT) or nitrogen supplementation would be necessary to treat rheumatoid cachexia (12).

Accumulating evidence supports the significant clinical benefit of resistance training in patients with RA [reviewed in Ref. (28)]. Regular training not only increased muscle strength but also improved disease activity, pain, Health Assessment Questionnaire (HAQ) scores, and walking speed in RA patients (28, 39–45). For example, Marcola (44) and Lemmey et al. (45) reported that PRT for a period of 12 and 24 weeks, respectively, increased muscle mass while reducing fat mass. Lemmey et al. also reported improvements in physical performance, while levels of muscle IGF-1 and IGF-binding protein (IGFBP)-3 in muscle increased (45).

Besides the resistance training, endurance training (ET) is also important to improve insulin sensitivity and body composition (46). In a report by Mikkelsen et al. (47), it was revealed that life-long endurance exercise was associated with lower levels of circulating CRP and IL-6 levels, and also with a greater muscle area compared to untrained controls, indicating potential anti-inflammatory effect of ET. In fact, the combination of resistance training and ET has been demonstrated to increase lean body mass and to decrease body fat in RA patients (48, 49). These findings suggest the therapeutic potential of these physical therapies in RA and other chronic inflammatory conditions.

### Other approaches

α-Melanocyte-stimulating hormone (α-MSH) is a proopiomelanocortin (POMC)-derived neuropeptide that exerts anorexigenic and anti-inflammatory properties (50). Recently, Gómez-SanMiguel et al. (7) reported that systemic administration of α-MSH to arthritis model mice resulted in improved food intake and higher BW gain with decreased arthritis signs. The authors found that increase in hypothalamic expressions of COX-2, IL-1β, POMC, and Agouti-related protein (a stimulator of appetite) mRNA were prevented by α-MSH administration in the arthritic mice. α-MSH treatment also decreased expressions of E3 ubiquitin ligases, which are markers of muscular atrophy. Thus peripheral α-MSH treatment is suggested to be an anti-cachectic strategy in arthritis.

Other approaches such as anabolic steroids or recombinant human GH have been proposed, however, there is no evidence to show their safety and efficacy (28).

## Concluding Remarks

**Figure 1:**
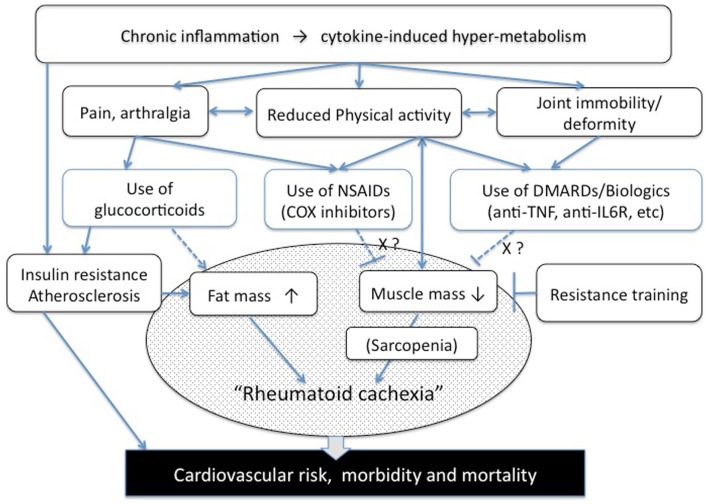
**Pathogenesis of rheumatoid cachexia (a hypothesis)**. NSAIDs, non-steroidal anti-inflammatory drugs, COX, cyclooxygenase; DMARDs, disease-modifying anti-rheumatic drugs.

Recent progress in anti-rheumatic therapies has made “classic” cachexia less frequent among RA patients. Nevertheless, a different form of abnormal metabolism, “rheumatoid cachexia,” has emerged, that may promote comorbid risks and deterioration in physical activity in RA patients (Figure 1).

To overcome the muscle loss and fat deposit in RA, it will be essential to first establish a consensus regarding definition of this disorder. Possible overlap with sarcopenia should be also noted. Further, tight control of disease activity, along with physical exercise and appropriate nutritional management, should be recommended to improve energy expenditure and reduce insulin resistance in RA patients.

## Conflict of Interest Statement

The author declares that the research was conducted in the absence of any commercial or financial relationships that could be construed as a potential conflict of interest.

## Supplementary Material

The Supplementary Material for this article can be found online at http://www.frontiersin.org/Journal/10.3389/fnut.2014.00020/abstract

Table S1 **Reported changes of indices of body composition in RA patients**. References: (6, 8, 10, 15, 16, 18, 20, 24, 43–50). Disease Activity Score-28 (51) of the tested patients were 2.6–6.39 and the Health Assessment Questionnaire (52) score was around 0.22–1.95. BCM, body cell mass; FFM, fat-free mass; LBM, lean body mass; AMA, arm muscle area, TEE: total energy expenditure, REE: resting energy expenditure. Biologics: TNF, TNFα inhibitors; IL-6, IL-6 receptor antagonist; CD20, CD20 inhibitor; ↑, increased; ↓, decreased; →, no change compared with the control group, respectively. *Values shown are at the patient’s first visit (baseline).
